# Multispacer typing of *Rickettsia* isolates from humans and ticks in Tunisia revealing new genotypes

**DOI:** 10.1186/1756-3305-6-367

**Published:** 2013-12-31

**Authors:** Abir Znazen, Fatma Khrouf, Nihel Elleuch, Dorra Lahiani, Chakib Marrekchi, Youmna M’Ghirbi, Mounir Ben Jemaa, Ali Bouattour, Adnene Hammami

**Affiliations:** 1 Laboratory of Microbiology, Research Laboratory “MPH”, Habib Bourguiba University Hospital of Sfax, Sfax, Tunisia; 2 Laboratory of entomology, Pasteur Institute, Tunis, Tunisia; 3 Infectious diseases department, Hedi Chaker University Hospital of Sfax, Sfax, Tunisia

**Keywords:** *Rickettsia*, Multispacer typing, Intergenic spacers, Vectors, Spotted fever rickettsioses, Tunisia

## Abstract

**Background:**

Rickettsioses are important remerging vector born infections. In Tunisia, many species have been described in humans and vectors. Genotyping is important for tracking pathogen movement between hosts and vectors. In this study, we characterized *Rickettsia* species detected in patients and vectors using multispacer typing (MST), proposed by Founier *et al.* and based on three intergenic spacers (dksA-xerC, rmpE- tRNA^fMet^, mppA-pruC) sequencing.

**Methods:**

Our study included 25 patients hospitalized during 2009. Ticks and fleas were collected in the vicinity of confirmed cases. Serology was performed on serum samples by microimmunofluorescence using *Rickettsia conorii* and *Rickettsia typhi* antigens. To detect and identify *Rickettsia* species, PCR targeting *ompA*, *ompB* and *gltA* genes followed by sequencing was performed on 18 obtained skin biopsies and on all collected vectors. *Rickettsia* positive samples were further characterized using primers targeting three intergenic spacers (dksA-xerC, rmpE- tRNA^fMet^ and mppA-purC).

**Results:**

A rickettsial infection was confirmed in 15 cases (60%). Serology was positive in 13 cases (52%). PCR detected *Rickettsia* DNA in four biopsies (16%) allowing the identification of *R. conorii* subsp *israelensis* in three cases and *R. conorii* subsp *conorii* in one case. Among 380 collected ticks, nine presented positive PCR (2.4%) allowing the identification of six *R. conorii* subsp *israelensis*, two *R. massiliae* and one *R. conorii* subsp *conorii*. Among 322 collected fleas, only one was positive for *R. felis. R. conorii* subsp *israelensis* strains detected in humans and vectors clustered together and showed a new MST genotype. Similarly, *R. conorii* subsp *conorii* strains detected in a skin biopsy and a tick were genetically related and presented a new MST genotype.

**Conclusions:**

New *Rickettsia* spotted fever strain genotypes were found in Tunisia. Isolates detected in humans and vectors were genetically homogenous despite location differences in their original isolation suggesting epidemiologic circulation of these strains.

## Background

*Rickettsiae* are Gram negative obligate intracellular rods belonging to the subgroup of alpha *Proteobacteriae*. These bacteria are closely related to arthropods that act as their vectors and reservoirs [1]. After transmission through a tick or flea bite, some pathogenic species cause polymorphic clinical features, essentially eruptive fever associated or not to inoculation eschar (tache noire) or isolated fever. A total of 28 species are validated into the genus *Rickettsia*, among which approximately 20 are recognized as human pathogens. Formerly, the classification of *Rickettisae* was based on serology and divided the genus into two sero-groups: typhus group and spotted fever group (SFG). Molecular and phylogenetic analyses classified the genus *Rickettsia* into at least three groups: SFG, typhus group, the *R. bellii* group (ancestral) [2,3]. Rickettsioses are considered to be important emerging vector born infections of humans worldwide. In Tunisia, *R. conorii* subsp *conorii*, the agent of Mediterranean spotted fever (MSF), was previously thought to be the unique species causing spotted fever rickettsiosis [4]. In recent years, many studies based on both serological and molecular techniques described a variety of species causing rickettsioses. Thus, *R. conorii*, *R. typhi, R. aeschlimannii* and *R. felis* were characterized by serology [5,6]. Using molecular methods, *R. conorii* subsp *conorii* and *R. conorii* subsp *israelensis* were detected in humans [7,8], *R. monacensis* and *R. helvetica* in *Ixodes ricinus*[9] and recently *R. massiliae* in *Rhipicephalus sanguineus*[10].

Molecular typing of infectious agents is important since it provides a better understanding of ecological niches and the spread of microorganisms. In rickettsiology, the bacterial dynamic between the bacterium, vectors and hosts is not completely studied. The genotyping of strains detected in human samples and in arthropods could help further our understanding of the circulating strains and identifying hypervirulent strains. For *Rickettsia* genus, Fournier *et al*. proposed a multispacer typing (MST) combining three spacers to distinguish rickettsial genotypes [11]. Indeed, intergenic spacers were shown to be better conserved and less submitted to selection pressure in intracellular bacteria. Herein, we aim to characterize rickettsial species detected both in humans and in vectors, using the multispacer typing method.

## Methods

### Patients

Our study was conducted during 2009 at the Infectious Diseases Department of Hedi Chaker University of Sfax Tunisia. Included patients were suspected to have rickettsial infection on the basis of clinical presentation (fever associated to cutaneous rash) and epidemiologic features (exposition to ticks and or fleas with or without a history of bite). All the subjects provided informed consent. The study was approved by the Habib Bourguiba University hospital ethics committee. Skin biopsies performed on the cutaneous rash using a punch (of 4 mm diameter) and serum samples were collected. The skin biopsies were stored at −80°C until their use.

### Tick and flea collection

Ticks and fleas, feeding on domestic animals, were collected in the vicinity of households with serologically or clinically confirmed human rickettsiosis cases from July to October 2009 in Sfax. They were manually collected from dogs, sheep and goats. All specimens were identified to species level using appropriate taxonomic keys [12,13]. All ticks and fleas were stored in 70% ethanol at room temperature until DNA extraction.

### Serology

Serology was performed by microimmunofluorescence assay using *R. conorii* and *R. typhi* antigens provided by the “Unité des Rickettsies” in Marseille France as described previously [14]. Titers higher than 1:32 for IgM and 1:128 for IgG were considered positive.

### DNA extraction

For skin biopsies and collected arthropods, DNA extraction was performed using QIAamp® DNA tissue extraction kit (Qiagen, Hilden, Germany) according to manufacturer’s instructions. DNA extracts were stored at −20°C until their use.

### PCR amplification and sequencing

To identify species detected in skin biopsies and ectoparasite vectors, *ompA*, *ompB* and *gltA* genes were amplified and sequenced using primers previously reported [15,16]. Molecular typing was performed for samples identified by at least two of the three genes listed above. Primers proposed by Fournier *et al*. were used to amplify three spacers: dksA-xerC, rpmE- tRNA^fMet^ and mppA-purC [17].

All PCR reactions were performed in a 50 μl reaction mixture containing 1 pmol of each primer, 200 μM(each) dATP, dGTP, dCTP and dTTP (Takara), 0.75 U Taq polymerase (ExTaq, TAKARA), 1X Taq buffer and 2 μl extracted genomic DNA. Amplification was carried out in a DNA thermocycler (Applied Biosystems) under the following conditions: 4 min of initial denaturation at 94°C, then 35 cycles of 94°C for 1 min, Tm°C for 0:30 sec and 72°C for 0:30 sec. The amplification was completed by holding for 7 min at 72°C to allow complete extension of the PCR products. In each PCR, *R. montanensis* was included as positive control and water as negative control. PCR products were visualized by ethidium bromide staining after electrophoresis in a 1.5% agarose gel and their sizes were estimated by comparison with a molecular mass standard (100 pb plus DNA ladder; Promega). The PCR products were purified using Quick-PCR Purification Kit (Qiagen, Hilden, Germany) as described by the manufacturer. Sequencing reactions were performed in the DNA Engine Tetrad 2 Peltier Thermal Cycler (BIO-RAD) using the ABI BigDye® Terminator v3.1 Cycle Sequencing Kit (Applied Biosystems), following the protocols supplied by the manufacturer. Each sequencing reaction was repeated at least twice in both the forward and reverse directions before being accepted for analysis.

### Sequence analysis and construction of phylogenetic tree

The obtained sequences of all amplicons were assembled using Bioedit software. *OmpA*, *ompB* and *gltA* sequences were blasted to Genbank to obtain identification at species level.

For MST, sequences of each spacer studied were blasted to GenBank and compared to reference strains showing the maximum identity. The accession number of the Reference strains showing 100% identity was referred to tables reported by Fournier *et al.* to determine the genotype [11]. MST genotypes were defined as unique combinations of the three spacers’ genotypes.

To infer relationships between the Tunisian isolates and other reference strains published in GenBank, three phylogenetic trees were constructed using the neighbor joining method (NJ) [18], the unweighted pair group method with arithmetic mean (UPGMA) [19] and the maximum parsimony method [20] within the MEGA 5.2 software. The most consistent method that gives the better outcome was adopted.

## Results

### Diagnosis of rickettsial infection in patients

During 2009, 25 patients were included in our study. Considering results of serology and PCR, diagnosis of rickettsial infection was made in 15 cases (60%) including 6 men and 9 women. The patients mean age was 43 years (17–80). All of our cases occurred between June and October. Serology was positive in 13 cases (52%). Sera reacted with *R. conorii* antigens in ten cases, with *R. typhi* antigens in 2 cases and showed cross reactions between the two groups in one case. Skin biopsies were performed in 18 cases. Rickettsial DNA was detected in 4 biopsies (16%). Identified *Rickettsia* species are listed in Table 1. *Rickettsia conorii* subsp *israenlensis* was detected in three cases with a sequence homology of 99% for *ompB* gene and 100% for *ompA* gene [GenBank: AF123712 and AY197564, respectively]. *Rickettsia conorii* subsp *conorii* was detected in one case with a sequence homology of 100% for *ompA* gene and 100% for *gltA* gene [GenBank: AE006914].

**Table 1:** ***Rickettsia *** species detected in Tunisian patients and in vectors and their spacer genotypes*

**N°**	**Source**	**Locality**	**Date**	**Species**	**dksA-xerC**	**rpmE-tRNA**^**fMET**^	**mppA-purC**
P45	Skin biopsy	Hzag	09-12-2009	*R. conorii* subsp *conorii*	B^a^	KF500414^1^	B^b^
P73	Skin biopsy	Menzel chaker	09-16-2009	*R. conorii* subsp *israelensis*	S^c^	C^d^	D^e^
P75	Skin biopsy	Menzel chaker	09-28-2009	*R. conorii* subsp *israelensis*	S	C	D
P77	Skin biopsy	Sidi bouzid	09-30-2009	*R. conorii* subsp *israelensis*	S	C	D
F220	*C. felis*	Kerkennah	30-07-2009	*R.felis*	AB^f^	P^g^	-
T116	*Rh. sanguineus*	Boujarbou	07-22-2009	*R. massiliae*	AE/CP003319^h^	EU250277/CP003319^i^	EU250278/CP003319^j^
T317	*Rh. sanguineus*	Djebeniana	07-22-2009	*R. conorii* subsp *israelensis*	S^k^	C^l^	D^m^
T322	*Rh. sanguineus*	Djebeniana	07-22-2009	*R. conorii* subsp *israelensis*	S	C	D
T323	*Rh. sanguineus*	Djebeniana	07-22-2009	*R. conorii* subsp *israelensis*	S	C	D
T328	*Rh. sanguineus*	Djebeniana	07-22-2009	*R. conorii* subsp *israelensis*	S	C	D
T330	*Rh. sanguineus*	Djebeniana	07-22-2009	*R. massiliae*	-	EU250277/CP003319	EU250278/CP003319
T332	*Rh. sanguineus*	Djebeniana	07-22-2009	*R. conorii* subsp *israelensis*	S	C	D
T336	*Rh. sanguineus*	Djebeniana	07-22-2009	*R. conorii* subsp *israelensis*	S	C	D
T421	*Rh. sanguineus*	Kerkennah	07-30-2009	*R. conorii* subsp *conorii*	B^n^	KF539830^2^	B

Table legend:

*Spacer genotypes are defined according to Fournier *et al*. database. If the spacer sequence showed a similarity less than 100% to those present in this database, the Genbank accession number of the sequence with 100% similarity or that of the new submitted sequence (1,2) is noted.

-: Amplification or sequencing failed.

Accession Numbers of submitted sequences : ^a^KF500412, ^b^KF500416, ^c^KF500411, ^d^KF500413, ^e^KF500415, ^f^KF539824, ^g^:KF539828, ^h^KF539823, ^i^KF539827, ^j^KF539831, ^k^KF539825, ^l^:KF539829, ^m^KF539832, ^n^KF539826.

### Detection of *Rickettsia* DNA in vectors

All collected ticks were identified as *Rhipicephalus sanguineus*. Among 380 tested ticks, nine were positive. Species detected are listed in Table 1. Only one tick (0.3%) was positive to *Rickettsia conorii* subsp *conorii* with a sequence homology of 100% for *ompA* and *gltA* genes [GenBank: AE006914]. Two ticks (0.5%) were infected by *Rickettsia massiliae* Bar 29 with 100% similarity for *ompA* gene [genbank U43792] and *gltA* gene [genbank U59720]. *Rickettsia conorii* subsp *israelensis* was detected in 6 ticks (1.5%). Three specimens were positive with 100%, 100% and 99% similarity to *ompA* [GenBank: AY197564], *ompB* [GenBank: AF123712] and *gltA* [GenBank: EF177484] genes, respectively. The remaining three ticks showed positivity for only two genes (*ompA* and *gltA*) with 100% similarity. All the 322 tested fleas were identified as *Ctenocephalides felis*. Only one (0.3%) was positive for *ompB* and *gltA* and the sequence analysis showed 100% identity to *Rickettsia felis* [GenBank: CP00053].

### Multispacer typing

**Figure 1:**
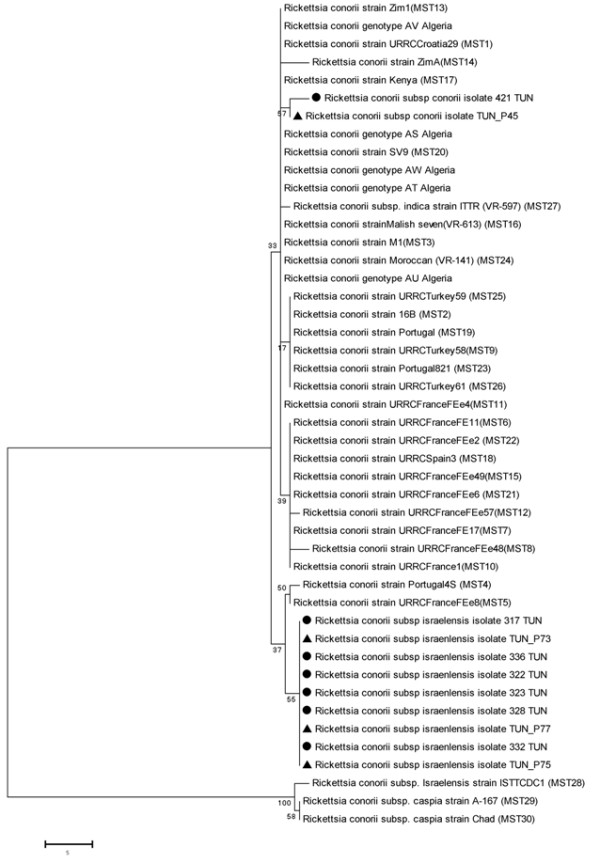
**Phylogenetic tree of MST genotypes of *Rickettsia conorii *isolates from humans and ticks, in Tunisia.** The tree was constructed using the Neighbor joining method based on 3 concatenated intergenic spacer sequences in 34 MST genotypes. Bootstrap values (100 replicates), shown in MEGA5.2 software, were used for phylogenetic analysis. Filled triangles indicate *R. conorii* strains detected in the skin biopsies of Tunisian patients in this study. Filled circles indicate *R. conorii* strains detected in ticks.

All *R. conorii* subsp *israelensis* isolates exhibit the same genotype for the three spacers (Table 1). Considering the combination of the three spacer’s type, a new MST genotype for Israeli spotted fever (ISF) strains was identified. Sequences of dksA-xerC, rpmE-tRNA and mppA-purC were submitted to Genbank with obtained accession numbers [KF500411, KF500413, KF500415, respectively] for a skin biopsy and [KF539825, KF539829, KF539832, respectively] for a tick. Adopted phylogenetic tree is presented in Figure 1. Our *R. conorii* subsp *israelensis* isolates, detected both in humans and ticks, were clustered together. *R. conorii* subsp *conorii* detected in a skin biopsy and a tick showed a new genotype for the spacer rpmE-tRNA^fMET^ [Genbank: KF500414, KF539830, respectively]. These two strains showed a new MST genotype and were clustered together (Table 1, Figure 1).

The two *R. massiliae* strains detected in ticks hold identical sequences as the strain *R. massiliae* AZT80 for the three spacers (CP003319). It is noteworthy that dksA-xerC amplification was successful for only one isolate (T116). For the *R. felis* strain, rpmE-tRNA^fMET^ and dksA-xerC spacers showed 100% identity to *R. felis* URRWXCa12 strain (MST genotype 43). However, mppA-purC amplification attempt was negative (Table 1).

## Discussion

Rickettsioses are endemic worldwide. Many epidemiological features of these infections need to be clarified. Genotyping of human and vector strains could help to identify circulating strains. Actually, many techniques are used for identification and genetic characterization of bacterial isolates. Using multispacer typing we characterized different *Rickettsia* species in specimens from ticks and humans from Tunisia.

Tracking the movement of causative organisms between animals and humans allows the evaluation of the risk of human infectious diseases [21,22]. In the public health setting, molecular typing of infectious agents is important for the study of bacterial population dynamics, the understanding of the ecological niches occupied by specific pathogen types in the environment, the spread of pathogens in outbreaks, the detection of disseminated antibiotic resistant strains in managed care facilities, the identification of hypervirulent strains, and the monitoring failures in live vaccination programs [23,24]. Molecular typing schemes based on the sequencing of intergenic regions have been developed. MST is a nucleotide sequencing based on genotyping method that uses highly variable intergenic spacers as typing markers. It is the most suitable genotyping procedure for evaluating the population structure of intracellular bacteria. Thus, it was developed for *Bartonella* and *Coxiella*[25,26]. This method was shown to be discriminatory not only for *Rickettsia conorii* but also for other *Rickettsia* species. When first described by Fournier *et al.*, MST identified 27 genotypes among 39 *R. conorii* studied strains [11]. The method was then applied to 22 *R. conorii* strains detected in Algeria and 7 genotypes could be differentiated [27]. Using this MST, 15 rickettsial isolates obtained from humans or ticks over a 26-year period in various areas of China were demonstrated to belong to *R. sibirica* subsp*. sibirica*. In Russia, also, tick born rickettsioses was confirmed to be caused by a *R. heilongjangensis* strain different from the prototype strain HLJ054 [28].

We were able to genotype *Rickettsia* species detected in our specimens directly without being cultured. In fact, the MST genotyping is PCR based. Of note, none of our samples showed nonspecific amplification and all amplicons provided a single band on the electrophoresis profile. In the study of Wenjun *et al.* the MST genotyping was also applied directly to specimens [27].

Among 20 analyzed skin biopsies, *R. conorii* subsp *conorii* was detected in only one patient while *R. conorii* subsp *israelensis* was detected in three patients. For two patients with Israeli spotted fever (ISF), epidemiologic and clinical characteristics were previously reported [8]. In ticks also, *R. conorii* subsp *israelensis* was more frequently detected than *R. conorii* subsp *conorii.* Contrary to the previously adopted hypothesis, that MSF caused by *R. conorii* subsp *conorii* is the most common rickettsioses in Tunisia, in our study *R. conorii* subsp *israelensis* was the most frequent agent detected in both humans and ticks. This could suggest that cases of ISF occurring during 2009 are epidemic. In fact, the phylogenetic tree showed that human and tick isolates were genetically homogenous despite the differences in location of their original isolation (Table 1). Moreover, ISF isolates were grouped in a separate cluster and presented a new MST genotype. The obtained genotype showed a genetic diversity with the single ISF strain included in the study of Fournier *et al. *[17]. The two genotypes differed in two spacers. As previously reported [8], one of our patients (P75) was suspected to be infected in Libya, suggesting the occurrence of the new genotype in other North African countries. Nevertheless, no ISF strain has been reported until now from neighboring countries. Further investigations are needed to define the distribution of this species and its genotypes in North Africa. Previously, we speculated that strains circulating in Libya could be more invasive since the patient (P75) presented a more severe disease. Boillat *et al*. also reported a case of a severe form of ISF with a fatal outcome of a patient suspected to be infected in Libya [29]. However, the strain detected in patient P75 was genetically homogenous to the other strains. Furthermore, in Portugal, Vitorino *et al*. found no correlation between virulence of ISF strains and the dksA-xerC profile [30].

*R. conorii* subsp *conorii* strains detected in a skin biopsy and a tick clustered together suggesting the circulation of this strain between humans and vectors. This strain presented a new rpmE-tRNA^fMet^ genotype and thus a new MST genotype, different from that previously reported in Tunisia [27]. In fact, Wenjun *et al.* studied the phylogenetic position of 61 *R. conorii* subsp *conorii* isolates, and reported MST genotype 3 in many Mediterranean regions (Algeria, Tunisia, Spain and France). In this study, seven genotypes among 22 skin biopsies were identified, revealing heterogeneity in circulating species in Algeria [27]. Our findings emphasize the distribution of varying genotypes of *R. conorii* in North Africa.

Infections caused by a variety of *Rickettsia* species have been previously described in Tunisia. *R. felis*, was diagnosed in many human cases by serology from our region and from the centre of Tunisia [5,6]. In our work, the DNA of the bacteria was detected in a flea. The strain found was similar to wild type (URRWXCa12) for two spacers. However, amplification of the spacer rpmE-tRNA^fMet^ failed. This could be explained by the direct amplification from specimens without culturing strains. Similarly to other north African countries (Morocco and Algeria) [31,32], *R. massiliae* was detected by PCR in *Rhipicephalus sanguineus* in Tunisia. This species was recently found in ticks analyzed by reverse line blot Assay [10]. MST genotypes of our strains were genetically homogenous with the *R. massiliae* AZT80 strain. This strain was first described in Spain as *Rickettsia massiliae* strain Bar29 [33] then in Arizona [34], which suggests its ubiquity.

## Conclusions

Our study reveals new genotypes of *Rickettsia* species detected in humans and in vectors using multispacer typing, despite the limited number of specimens. Our findings suggest a special epidemiologic situation in 2009 with more frequent infections with ISF strains and reveal a risk of acquiring a variety of spotted fever infections in Tunisia. Moreover, our data clarify the epidemiologic status of rickettsial infection and underline their specificities. The detection of the same genotype in both patients and vectors emphasizes the role of *Rh. sanguineus* in transmission of ISF. To demonstrate the endemicity of the specific genotype of ISF *Rickettsia* detected, expanded studies in time and in locations are needed. Further, systematic notifications of cases of rickettsioses are necessary to explore the identification and genotypes of spotted fever *Rickettsia* in vectors and clinical specimens in Tunisia. A Continuous surveillance of SFG *Rickettsia* and their genotypes is important to understand the epidemiology of Rickettsial infection. The integration of such data in an international database could help to understand the circulation of strains and their emergence or reemergence.

## Competing interests

The authors declare that they have no competing interests.

All this work was financed by the research laboratory “MPH, Habib Bourguiba University Hospital” Sfax, and the laboratory of epidemiology and veterinary microbiology, Pasteur institute, Tunis, Tunisia.

## Authors’ contributions

AZ and FK: collected samples, performed molecular and data analysis and drafted the manuscript; NE: participated to DNA extraction and molecular analyses; DL, CM and MBJ: collected samples and clinical data; YM: coordinated sample collection and participated to data analysis; AB: collected samples, supervised the study and reviewed the manuscript. AH: supervised the study and reviewed the manuscript. All authors read and approved the final manuscript.

## Authors’ information

Dr Abir Znazen works as a Professor’s assistant at The Faculty of Medicine of Sfax Tunisia since 2005. She is involved in the laboratory diagnosis of infectious diseases caused by intracellular bacteria. Previously, she reported *Rickettsia felis* and *R. conorii* Israeli spotted fever infections in Tunisia. Actually, she continues working on the epidemiology of these infections and tries to improve the diagnosis of these infections in his laboratory.

Fatma Khrouf has been a biological engineer since 2008. Currently, she is a PHD student in Pasteur institute in Tunisia. Her research interest is vectors and vector born diseases mainly Rickettioses.
